# The Army Medical Reserve Corps

**Published:** 1908-11

**Authors:** 


### BUFFALO MEDICAL JOURNAL.

A Monthly Review of Medicine and Surgery.

*EDITOR:*

WILLIAM WARREN POTTER, M. D.

All communications, whether of a literary or business nature, books for reviewand exchanges, should be addressed to the editor, 238 DELAWARE AVENUE, BUFFALO, N. Y.

VOL. LXIV. NOVEMBER, 1908. No. 4

### The Army Medical Reserve Corps.

AFTER years of effort on the part of the Surgeon General, the Medical Reserve Corps of the United States Army has been organised in compliance with the law passed at the last session of Congress, and commissions have been issued by President Roosevelt.

This is the final act in a struggle—and it has been a struggle against official indifference and Congressional stupidity—which has been waged by the medical journals of this country, almost without exception, in furtherance of the efforts of the Surgeon General to secure material for the up-building of the finest and best equipped medical corps of any army in the world, and to remove from official life a most humiliating figure, that of contract surgeon. The contract surgeons, in the main, able and capable men, by reason of their official designation were little more than mere conveniences and were looked down upon and ignored by all officialdom with the exception of the Surgeon General's office and the members of his staff. There is little use in reviewing the course of the fight which has been made for the establishment of the Medical Reserve Corps and for an increase in the numerical strength of the medical corps of the army. The course of the struggle has been noted from time to time in the JOURNAL and its final victory was announced a short time ago when Congress passed, and the President signed, the bill making provision for the recognition which had been so long demanded on behalf of the medical profession.

Beginning with an editorial in the JOURNAL calling attention to the anomalous position of the acting assistant surgeon during the Spanish war the agitation grew until it assumed national proportions and it is creditable to the medical profession at large to know that, with very few exceptions, there was unanimity of opinion regarding the rights of the medical officer who, while so designated, had few of the rights of an officer. As an acting assistant surgeon he performed all the duties of a commissioned officer except acting as a member of courts martial; he wore the uniform of the service; he acted on boards of survey; he signed and receipted for property; he was held responsible for all properties in his care; he took exactly the same oath of office that a commissioned officer swore to; he was in command of detachments of hospital corps men; he could not enlist men, but he could recommend their discharge on surgeon's certificate of disability; he went to the firing line with commissioned officers and enlisted men and he performed the duties of a soldier and a surgeon. Occasionally an acting assistant surgeon was killed or died of disease. The only difference between him and a commissioned officer was that he did not wear a sword and his collar ornaments were of silver instead of gold, and in the eyes of the majority of the line he was "only a contract." He was "only a contract" even when his official title was acting assistant surgeon, with the relative rank of a first lieutenant.

In spite of these known facts there was little difficulty in securing well equipped men for service when necessity arose. Probably this system might have been slower of eradication had not one of those acutely brilliant Washingtonian minds conceived the idea that the acting assistant surgeon was getting too much glory by being designated as an assistant surgeon and the title was changed to contract surgeon, and the collar ornaments ordered to consist of merely the letters "C. S." Then the medical journals and the profession began a systematic effort to further the plans of the Surgeon General. The increase in the personnel of the medical corps of the army was granted, much too small as it was, and with it went the provision for the establishment of the Medical Reserve Corps which is of inestimable importance when viewed from the standpoint of army medical efficiency. The bill creating the corps provides examination for entrance which is nearly the same as that for entrance into the medical corps of the army.

The first move on the part of the Surgeon General was to immediately transfer all the men serving as "contract surgeons" to the medical reserve corps and secure for them commissions. This was a simple matter being merely a question of transfer. With that cleared up came the work of establishing the real Medical Reserve Corps, the organisation of the nucleus of what will eventually be a thoroughly competent, well-equipped medical service which will not have its equal in any army in the world. Invitations were sent to some of the most prominent surgeons in this country to become members of the corps in an advisory capacity, and that many have accepted speaks well for the success of the corps. The complete list has not yet been made public, but commissions have been issued to Dr. Roswell Park of Buffalo, professor of surgery in the medical department of the University of Buffalo; Dr. J. C. DaCosta, professor of surgery in the Jefferson Medical College, of Philadelphia, and Dr. Frank P. Foster of New York City, editor of the New York Medical Journal. The next step in the organisation of the corps was the selection of men for commissions who had served as acting assistant surgeons and shown executive ability in army medical matters, or who had served as acting surgeons at posts and thus gained a knowledge of army routine.

Thus far has the work of organisation progressed and now to the corps will be added the younger members of the profession who wish to make army medicine their profession, and those who by reason of some decided qualification are fitted for special work in the event of emergency. With an organisation composed of experienced men and specialists in medicine and surgery, it is hard to conceive of an emergency which the Surgeon General of the Army cannot meet with comparative ease and promptness.

The disgraceful lack of preparedness which was demonstrated by the on-coming of the Spanish war will never be repeated. Here General Sternberg was plunged into a situation demanding a large number of surgeons. He had a woefully small medical corps for the work on hand and was compelled to employ a large number of acting assistant surgeons, men who, professionally well-qualified and competent, were wholly lacking in even a suspicion of technical detail. It is a monument to the steadfastness of the profession and to the versatility and executive ability of the then Surgeon General that the emergency was met as it was and with so little criticism in the face of official incapacity in other departments upon which depended the production of medical supplies at points of necessity.

So unprepared a period cannot again occur. Should war become a fact tomorrow, General O'Reilly will have a much larger medical corps at his command, and in addition could call upon men who had army experience, as well as a large number of commissioned officers well grounded in military medicine.

The details of the final organisation are not yet completed, but it is quite likely that the members of the reserve corps will be required to make reports to the Surgeon General's office in much the same manner that a commissioned officer on duty reports. The medical reserve corps officer should be furnished with the printed material concerning executive work of the department, in order that he may be kept in touch with changes in routine; he should be furnished with a copy of the medical manual and such other literature as is necessary for the proper preparation for his assuming charge of any branch of the medical work of the army. He is a commissioned officer; he is a medical man, and he only needs the finishing touches of the executive and so-called "paper work" to become a thoroughly well-equipped army medical officer.

There are great possibilities in this organisation; great possibilities for great good, if the material at hand is taken advantage of as undoubtedly it is the Surgeon General's intention. If the members of the corps are trained in army detail, they will become a most valuable adjunct to the regular medical corps. If the reserve corps is organised and allowed to vegetate as merely commissioned officers, their value in the event of sudden emergency would be little more than that of the acting assistant surgeon in the time of the Spanish war, when millions of dollars worth of Government property was placed in the care of medical men, who had not the slightest conception how to care or account for it.

The Medical Reserve Corps should be taught to report itself to the proper officer; it should be made thoroughly familiar with army medical matters and literature and correspondence, and so gradually brought into close touch with the Surgeon General's office; so far as possible, each member of the corps with the possible exception of those who act in an advisory capacity, should be given at intervals practical experience in executive work under the guidance of the post surgeon at the nearest army hospital. This may mean more or less of an inconvenience to some of the members of the corps, but the knowledge that he is fitting himself for actual, practical and satisfactory work in the event of emergency should more than counterbalance the minimum of inconvenience. By the adoption of some such measure of detail organisation, the medical reserve corps can be made an organisation flexible, smooth-working and ready for instant service. The fact that the medical reserve corps officer might not be called upon to do executive work is a secondary consideration; the fact that he is prepared to do it and competent to take charge of any branch of army medical work is the point to be achieved.

Commissions have been issued to Dr. Nelson W. Wilson, lecturer on genitourinary diseases in the University of Buffalo, who served as an acting assistant surgeon in the Spanish war, and to Dr. Edgar R. McGuire, lecturer on surgery in the University of Buffalo, who has served on several occasions as acting surgeon at Fort Porter during the absence of the post surgeon.

The medical reserve corps is uniformed the same as an officer of the medical corps, with the exception that the letters "R. C." are intertwined in the U. S. of the collar ornament.

AWARDS to successful competitors in the exhibition, which formed an essential part of the recent International Congress on Tuberculosis, were announced October 11, 1908, as follows:

For the best evidence of effective work in the prevention of tuberculosis since the last congress, in 1905, cash prizes of $500 each to the Women's National Health Association of Ireland and to the New York Charity Organisation Society; gold medals to the Swedish and Boston associations.

The prize of $1,000 for the best exhibit of an existing sanatorium for the treatment of curable cases was divided, $500 being given each to the White Haven (Penn.) Sanatorium and the Brompton Hospital Sanatorium, of Frimley, England; gold medals to the Doelitz Sanatorium, of Berlin, and the Adirondack Cottage Sanatorium, Saranac Lake, N. Y.

For a furnished house for the families of the working class, gold medals to Milton D. Morrill, of Washington, and José F. Toraya, of Cuba.

One thousand dollars to the Henry Phipps Dispensary, Baltimore, for the best exhibit of a dispensary for treatment of the tuberculous poor; gold medals to the Manhattan Tuberculosis Dispensary, of New York, and the Henry Phipps Institute, Philadelphia.

To the Brompton Hospital, of London, the $1,000 prize for the best exhibit of a hospital for the treatment of advanced pulmonary tuberculosis; gold medals to the Loomis Sanatorium, Liberty, N. Y., and the Massachusetts State Hospital, Tewkesbury, Mass.

The prize of $100 for the best educational leaflets, to the Pennsylvania Society for the Prevention of Tuberculosis, Philadelphia, and the Verein für Bekämpfung der Schwindsucht, Chemnitz and Umgebung; gold medals to Dr. O. D. Wescott, Denver; Dr. H. S. Goddall, New York, and George H. Kress, Los Angeles.

New York won the gold medal for the best exhibit sent in by the states illustrating effective organisation for the restriction of tuberculosis. A gold medal went to Germany for its national exhibit on this subject. For the best contribution to the pathological exhibit gold medals were presented to the United States bureau of animal industry and to England. Wisconsin won the gold medal for an exhibit of the best laws and ordinances in force in June, 1908, for the prevention of tuberculosis, while New York city won a gold medal for the best repressive municipal laws.

Among the other awards were special gold medals for various kinds of work in the campaign against tuberculosis to the United States Department of the Interior, the Government Printing

Office, Department of Commerce and Labor, Marine Hospital Service, Census Bureau, New York State Charities Aid Association, Sea Breeze Hospital, of Coney Island; Playground Association of America and the International Children's School Farm League, of New York city.

---

ELSEWHERE we publish the action of the commission created by the Buffalo Academy of Medicine, and of the committee appointed by the Medical Society of the County of Erie in reference to building a hospital for the care of contagious diseases, which should receive the considerate attention of every reader of the JOURNAL in Buffalo and Erie County. It is stated that the state department of health proposes to investigate the question, *inter alia,* of the inadequacy of Buffalo's facilities for the care of contagions. Better take action at once, Mr. Aldermen and Mr. Supervisors!

